# Silencing of *PMEPA1* accelerates the growth of prostate cancer cells through AR, NEDD4 and PTEN

**DOI:** 10.18632/oncotarget.3526

**Published:** 2015-03-30

**Authors:** Hua Li, Ahmed A. Mohamed, Shashwat Sharad, Elizabeth Umeda, Yingjie Song, Denise Young, Gyorgy Petrovics, David G. McLeod, Isabell A. Sesterhenn, Taduru Sreenath, Albert Dobi, Shiv Srivastava

**Affiliations:** ^1^ Center for Prostate Disease Research, Department of Surgery, Uniformed Services University of the Health Sciences, Bethesda, MD 20814, USA; ^2^ Urology Service, Walter Reed National Military Medical Center, Bethesda, MD 20814, USA; ^3^ Joint Pathology Center, Silver Spring, MD 20910, USA

**Keywords:** prostate cancer, PMEPA1, androgen receptor, NEDD4, enzalutamide

## Abstract

Androgen Receptor (AR) is the male hormone receptor and a nuclear transcription factor which plays a central role in the growth of normal and malignant prostate gland. Our earlier studies defined a mechanistic model for male hormone dependent regulation of AR protein levels in prostate cancer (CaP) cells through a negative feed-back loop between AR and PMEPA1, an androgen induced NEDD4 E3 ubiquitin ligase binding protein. This report focuses on the impact of *PMEPA1* silencing on CaP biology. *PMEPA1* knockdown accelerated the growth of CaP tumor cells in athymic nude mice. In cell culture models knockdown of *PMEPA1* resulted in resistance to AR inhibitors enzalutamide and bicalutamide. While, AR protein down regulation by NEDD4 was PMEPA1 dependent, we also noted a PMEPA1 independent downregulation of PTEN by NEDD4. In a subset of human CaP, decreased *PMEPA1* mRNA expression significantly correlated with increased levels of AR transcription target PSA, as a surrogate for elevated AR. This study highlights that silencing of *PMEPA1* accelerates the growth of CaP cells through AR, NEDD4 and PTEN. Thus, the therapeutic restoration of PMEPA1 represents a promising complementary strategy correcting for AR and PTEN defects in CaP. Statement of significance: Here we define that silencing of *PMEPA1* facilitates the growth of CaP cells and modulates AR through NEDD4 and PTEN. The restoration of *PMEPA1* represents a promising complementary therapeutic strategy correcting for AR and PTEN defects.

## INTRODUCTION

AR is a nuclear transcription factor that executes key function in normal and malignant growth of the prostate gland [1–5]. The tight control of AR signaling is critical in maintaining the homeostasis of the prostate gland. Malignancy of prostate still remains the most common cancer detected in American men and second leading cause of cancer related deaths [6, 7]. Cancer-associated dysfunctions of AR, such as aberrant activation due to mutations, amplification, splicing and cross talk with other pro-cancer signaling pathways contribute to prostate cancer (CaP) development and progression [1–5, 8]. In clinical practice, androgen ablation continues to be the corner stone for the treatment of advanced CaP [7, 9–11]. Unfortunately, androgen ablation treated CaPs eventually become treatment resistant, the stage of the disease known as castration resistant CaP (CRPC). Altered AR expression or function (gain or loss) resulting from genomic or non-genomic mechanisms associate with CRPC [1–5]. In contrast to other aspects of AR alterations, only a few studies have addressed how AR protein turnover is altered in CaP [12, 13]. A shorter half-life of AR was noted in the androgen responsive LNCaP cells as compared to its longer half life in the androgen refractory derivative C4–2B cells [14]. Early reports of the mechanisms of AR degradation showed that MDM2 E3 ubiquitin ligase targeted the AR through AKT mediated phosphorylation of MDM2 [15]. Our studies have implicated NEDD4 E3 ligase in AR degradation [16–19]. Studies have also described downregulation of AR by proteasome dependent degradation during mitosis suggesting AR as a licensing factor for DNA replication in CaP cells [20, 21]. Emerging data also suggests for the role of other E3 ubiquitin ligases (CHIP, SKP2, Siah2, RNF6) in the AR degradation or transcriptional activation [13]. A recent report showed that SPOP E3 ubiquitin ligase degrades AR protein and the recurrent genomic mutations of the *SPOP* noted in 5–10% of CaPs impair the SPOP mediated AR degradation [22, 23].

*PMEPA1* was originally identified by our laboratory as a prostate abundant, highly androgen induced gene that mapped to chromosome 20q13.31-q13.33 [16]. Human PMEPA1 protein exhibits 83% amino acid identity to the mouse Nedd4-bindng protein, N4wbp4 [16, 24]. *PMEPA1* is a direct transcriptional target of AR in CaP cells [18]. Further Investigations discovered a PMEPA1- AR negative feedback loop in the regulation of AR protein levels in CaP cells [19]. Members of the NEDD4 family of proteins are E3 ubiquitin ligases, which catalyze degradation of target proteins of physiologically important functions by the ubiquitin-prosteasome pathway [25, 26]. Initial evaluations of *PMEPA1* mRNA expression in matched normal and prostate tumor specimens suggested decreased expression of *PMEPA1* in two-third of CaP patients [17]. In contrast to CaPs, higher expression of *PMEPA1* has been noted in multiple solid tumors [27, 28]. Studies have also shown induction of *PMEPA1* expression by transforming growth factor-β (TGF-β) that was associated with colonocyte terminal differentiation [29]. Subsequent studies have defined that PMEPA1 inhibits TGF-β receptor 1 meditated signaling through a negative feedback loop by sequestering R-Smads [30]. Increased *PMEPA1* expression in breast and lung cancer may lead to inhibition of TGF-β signaling [31–33]. PMEPA1 has also been reported to promote the proliferation of AR negative CaP cells, PC3, through the Smad3–4/C-MYC/p21Cip1 pathway [34, 35]. Taken together, both reduced and increased *PMEPA1* expression may promote tumorigenesis through distinct cell signaling pathways in a given cellular background.

In this report, we present new findings on the cancer biologic properties of decreased *PMEPA1* expression. Loss or decreased *PMEPA1* expression in CaP contributes to accelerated cell growth through increased AR and NEDD4, decreased PTEN levels and confers resistance to AR inhibitors used in androgen ablation therapy.

## RESULTS

### Inhibition of *PMEPA1* promotes the growth of prostate cancer cells

We have examined the impact of *PMEPA1* depletion on tumor growth *in vivo*. We have characterized *PMEPA1*shRNA-LNCaP and *PMEPA1*shRNA-VCaP stable transfectants to assess the growth of prostate tumor cells *in vitro*. We observed enhanced cancer cell biologic features by monitoring cell growth, BrdU incorporation, cell plating efficiency, soft agar colony formation, growth response to androgen and cell cycle (Supplementary Figure 1).

**Figure 1:**
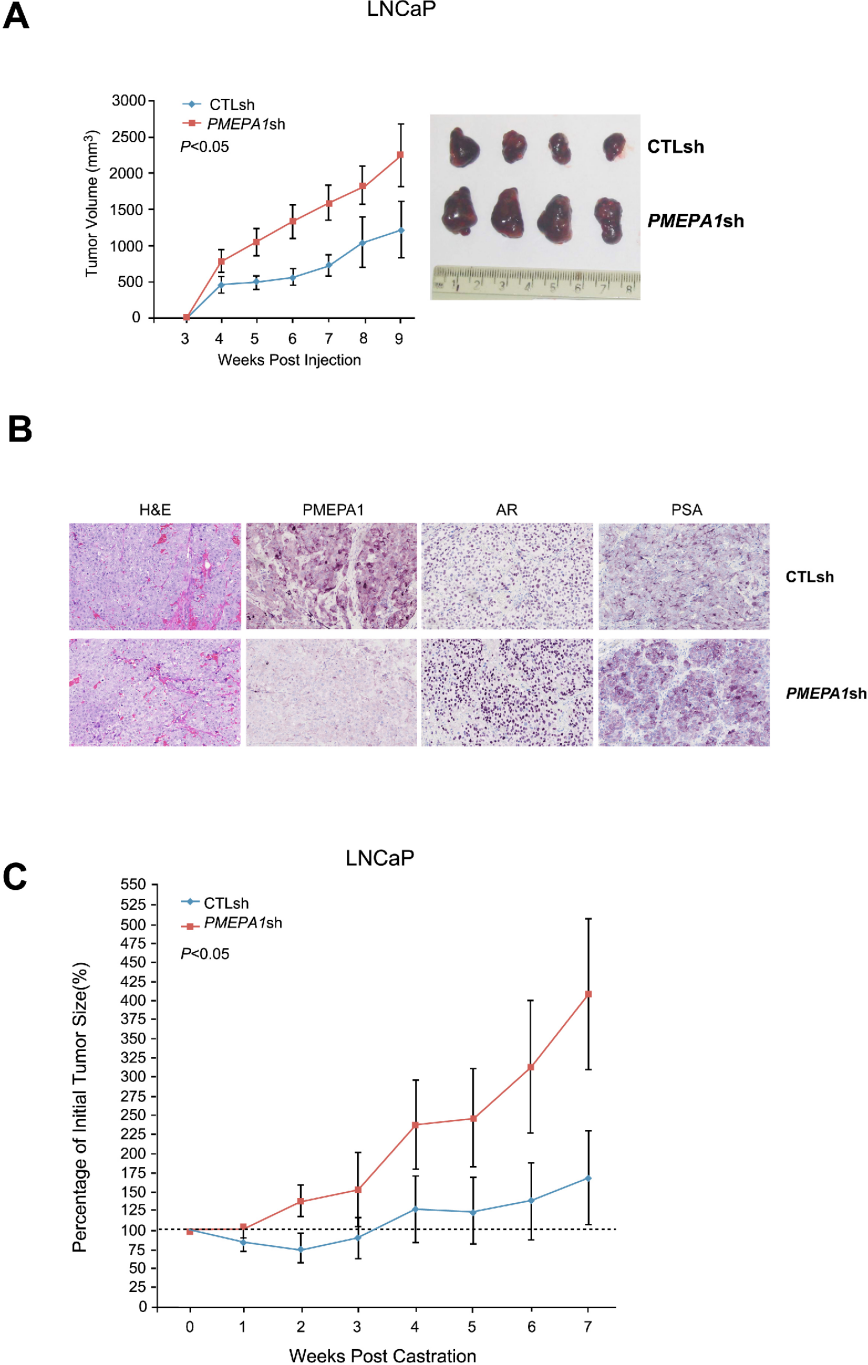
Inhibition of *PMEPA1* promotes the growth of prostate cancer cells *in vivo* **(A)** LNCaP cells harboring *PMEPA1*shRNA form larger volume of xenografts in athymic nude mice compared to control shRNA (CTLsh) LNCaP cells (graph and photograph of representative tumors; *p* < 0.05). **(B)** Elevated levels of AR and PSA are detected in tumors of *PMEPA1*shRNA expressing LNCaP cells by immunohistochemistry (representative IHC images at 20X magnification). **(C)** Castration attenuates the growth of LNCaP tumors *in vivo*, in contrast, *PMEPA1*shRNA harboring tumors continue to grow at accelerated rate (*p* < 0.05).

We evaluated the tumor growth characteristics of *PMEPA1*shRNA harboring LNCaP cells in athymic nude mouse tumor xenograft model. The *PMEPA1*shRNA-LNCaP and control shRNA-LNCaP cells began to form subcutaneous tumors at 4 weeks post-injection. The overall growth of *PMEPA1*shRNA-LNCaP derived tumors was significantly faster than control derived tumors (*p* < 0.05) (Figure 1A). At 9 week post-injection among 20 mice in each group, 18 mice formed measurable subcutaneous tumors in the *PMEPA1*shRNA-LNCaP group. In contrast, only 12 mice formed tumors in the control group. The tumor formation rate was 90% in the *PMEPA1*shRNA-LNCaP group and only 60% in the control. The average tumor volume was 2246.04 mm^3^ for *PMEPA1*shRNA-LNCaP and 1211.64 mm^3^ for the control. *PMEPA1*shRNA-LNCaP tumors expressed higher levels of AR and PSA proteins compared to control cells as assessed by immunohistochemistry (IHC) (Figure 1B and Table 1). The tumor size in the control group decreased 21% at two weeks post-injection in response to castration. In contrast, *PMEPA1*shRNA-LNCaP xenografts were refractory to castration. By seven weeks post-castration, the overall tumor size was four times larger than the initial tumor size (100%) in *PMEPA1*shRNA harboring xenografts (increased by 304%), in contrast to the control group (increased by 69%) (*p* < 0.05) (Figure 1C). Taken together, both *in vitro* and *in vivo* growth characteristics of *PMEPA1*shRNA harboring cells resulted in increased AR, gain of AR function and accelerated tumor growth under normal, as well as under castrate conditions.

**Table 1:** Higher expression level of AR and PSA were detected in *PMEPA1*shRNA-LNCaP xenografts with IHC staining

LNCaP xenografts	Area (%) of positive cells		
	PMEPA1	AR	PSA
CTLsh	37.13 ± 1.76	12.57 ± 3.32	7.02 ± 1.20
*PMEPA1*sh	2.86 ± 0.70	32.58 ± 1.46	17.65 ± 2.49
*P* value	<0.01	<0.01	<0.01

### *PMEPA1* depletion leads to resistance to AR inhibitors

**Figure 2:**
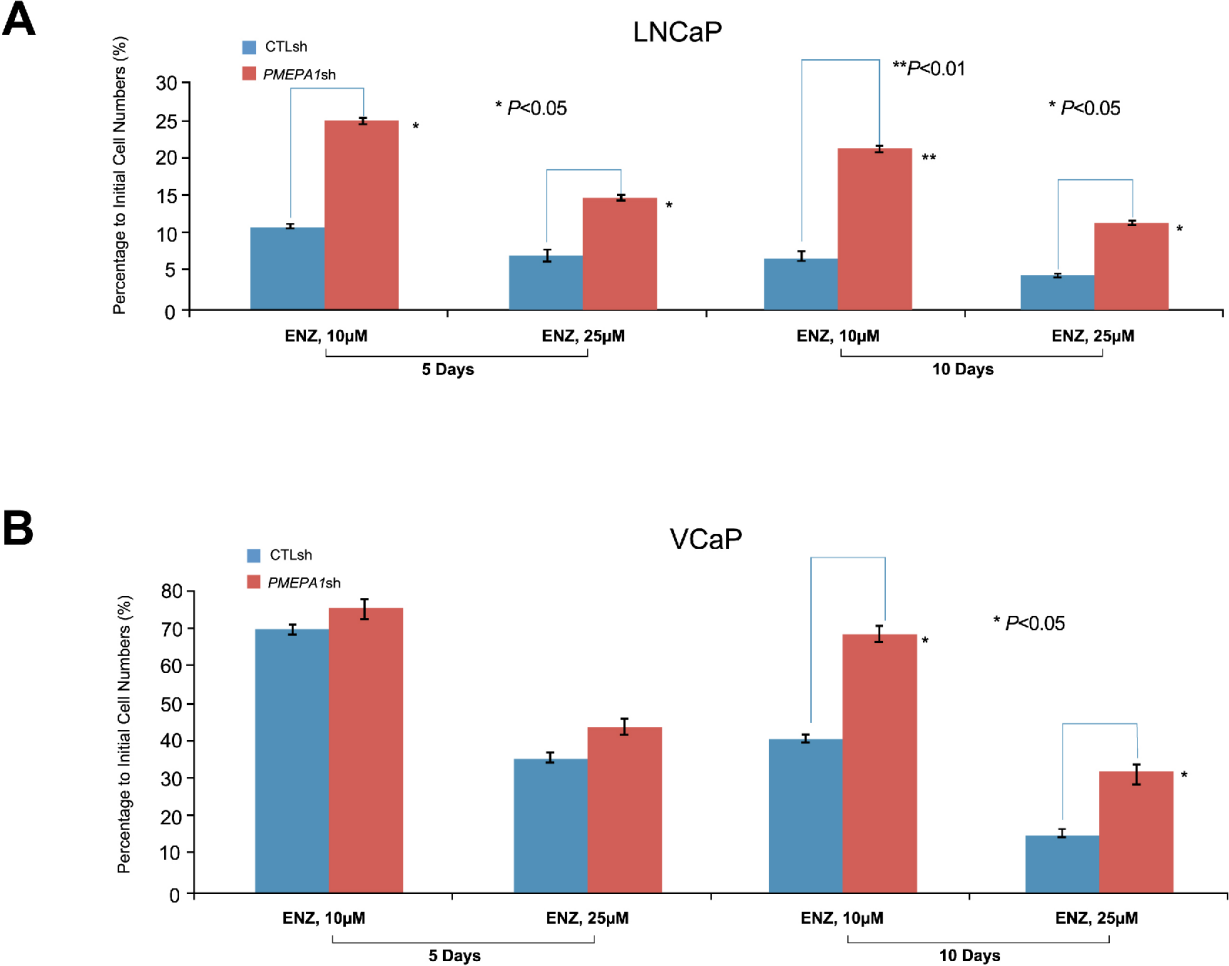
*PMEPA1* depletion leads to resistance to AR inhibitors **(A** and **B)** In contrast to control cells, increased percentages to initial cell numbers (2 × 10^5^ cells/6 cm dish) were detected in *PMEPA1*shRNA harboring LNCaP and VCaP cells in response to enzalutamide (ENZ) treatment at dosages of 10 or 25 μM for 5 or 10 days (*p* < 0.05).

To further investigate the role of decreased *PMEPA1* levels in enhancing tumor cell growth by gain of AR function, dose and time kinetic response to the AR inhibitors, enzalutamide and bicalutamide were assessed in cell growth assays. *PMEPA1* depletion conferred resistance to AR inhibitors in both LNCaP and VCaP cells (Figure 2A and 2B, Supplementary Figure 2A and 2B). Additionally, enhanced resistance to AR inhibitors was confirmed by BrdU incorporation, soft agar colony formation and cell plating efficiency assays (Supplementary Figure 2C–2E). Consistent with these observations, cell cycle analysis showed higher number of cells in S-phase and decreased rate of apoptosis in response to *PMEPA1* inhibition (Table 2 and Supplementary Figure 2F). The observed enhanced resistance to AR inhibitors in response to *PMEPA1* depletion was consistent with the observed castration resistance of *PMEPA1*shRNA harboring tumor xenografts. Taken together, these data suggest that loss or decreased *PMEPA1* levels confers resistance to AR inhibitors.

**Table 2:** Enzalutamide (ENZ) or bicalutamide (BIC) treated LNCaP cells showed higher percentage of S-phase and lower apoptosis rate in *PMEPA1*shRNA harboring cells

LNCaP cells	0 μM		BIC 25 μM		ENZ 25 μM	
	S-phase (%)	Apoptosis (%)	S-phase (%)	Apoptosis (%)	S-phase (%)	Apoptosis (%)
CTLsh	14.62	6.87	8.45	18.37	6.95	23.77
*PMEPA1*sh	19.24	4.12	13.62	9.11	12.11	11.22
*P* value	0.0022	0.0053	0.0168	0.007	0.023	0.0162

### *PMEPA1* silencing rescues AR inhibition in prostate cancer cells

**Figure 3:**
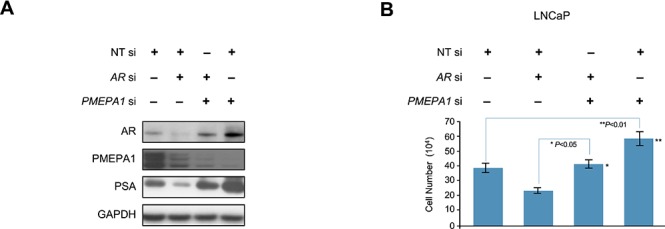
*PMEPA1* silencing rescues AR inhibition in prostate cancer cells **(A)** Western blot assay shows that *AR* siRNA (*AR* si) efficiently decreased protein levels of AR and PMEPA1 when compared to control (NT si). Co-transfection of *AR* and *PMEPA1* siRNA in LNCaP cells reversed effects of the *AR* depletion by siRNA on AR protein. **(B)** Cell counting assay reveals that *PMEPA1* siRNA (*PMEPA1* si) reversed the cell growth inhibition cause by *AR* siRNA in LNCaP cells (*p* < 0.01).

In hormone sensitive CaP cells, decreased AR expression levels results in reduced cell growth. However, under this condition, loss of *PMEPA1* may supersede the growth effect of AR inhibition by stabilizing AR at protein levels. Thus, we evaluated if inhibition of *PMEPA1* can restore CaP cell growth overriding the inhibition of AR. Indeed, co-transfection of *AR* and *PMEPA1* siRNAs in LNCaP cells reversed effects of *AR* depletion by siRNA on AR protein (Figure 3A). While parental LNCaP cells exhibited severe growth inhibition in response to *AR* siRNA, simultaneous knockdown of *PMEPA1* rescued the growth of cancer cells (Figure 3B). These data further supported that the observed enhanced cell proliferation in response to *PMEPA1* depletion was due to elevated AR protein levels and AR signaling.

### Disruption of AR and NEDD4 link by loss of *PMEPA1*

**Figure 4:**
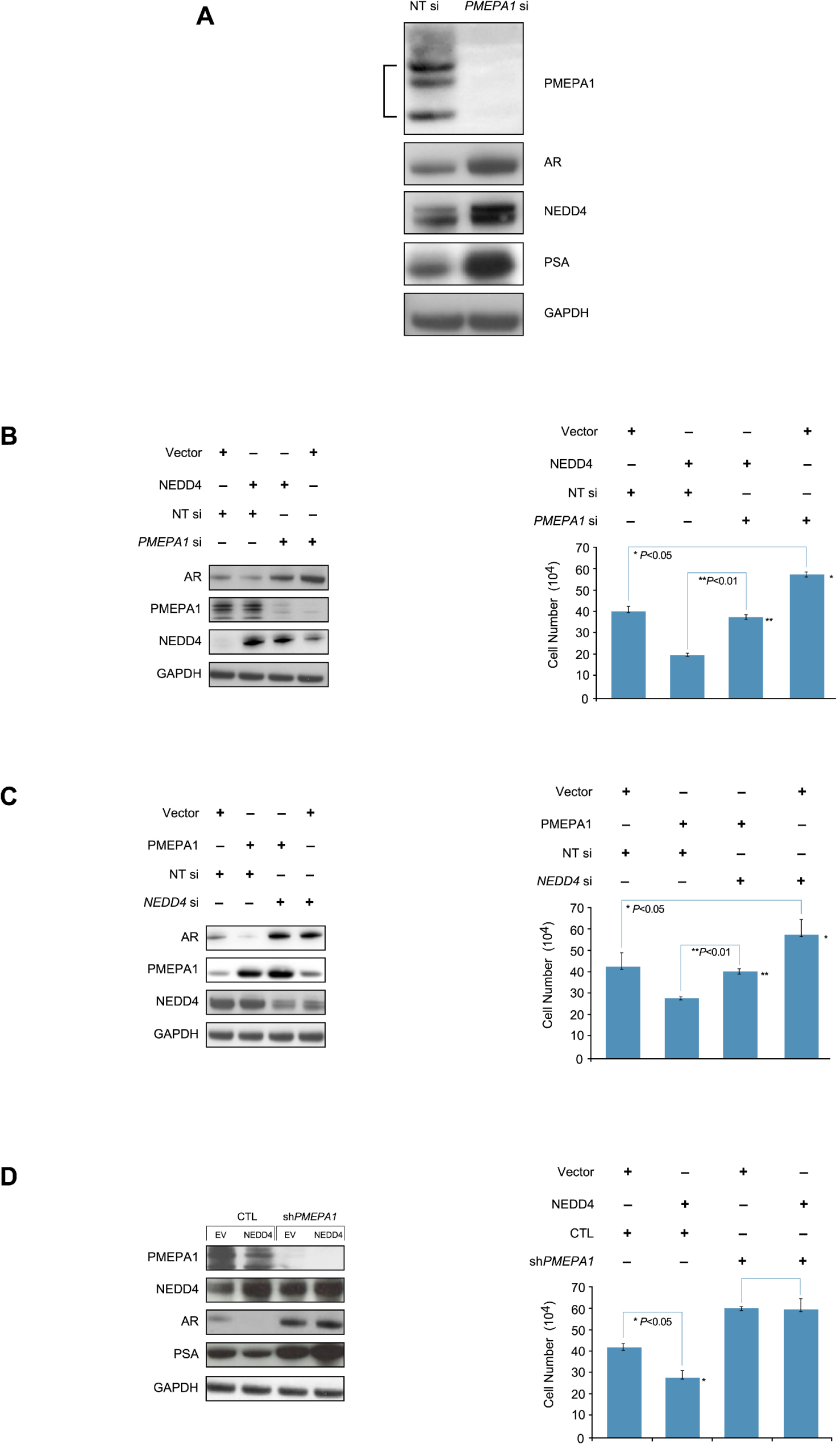
AR and NEDD4 link is disrupted by the loss of *PMEPA1* **(A)** *PMEPA1* knockdown increases protein levels of NEDD4, AR and PSA in LNCaP cells as shown by Western blot assay. **(B)** Knockdown of *PMEPA1* stabilizes AR (Western blot assay, left panel) and overrides the growth inhibitory effect of ectopically expressed NEDD 4 (cell counting assay, right panel; *p* < 0.01). **(C)** *NEDD4* siRNA stabilizes AR (Western blot assay, left panel) and reverses the cell growth inhibitory (cell counting assay, right panel) effect of PMEPA1 overexpression (*p* < 0.01). **(D)** Elevated expression of NEDD4 alone does not affect AR and PSA protein levels (Western blot assay, left panel) or cell growth (cell counting assay, right panel; *p* < 0.05) in *PMEPA1*shRNA harboring LNCaP transfectants.

It was intriguing to note simultaneous increase of NEDD4 and AR in response to *PMEPA1* knockdown (Figure 4A). This observation suggested that inhibition of *PMEPA1* may disrupt interaction between NEDD4 and AR. This observation was also supported by marked stabilization of AR in LNCaP cells harboring ectopically expressed NEDD4 in the background of *PMEPA1* knockdown (Figure 4B). Further, decreases in AR levels in response to ectopic *PMEPA1* expression were abolished by *NEDD4* knockdown in LNCaP cells (Figure 4C). As expected, the cell growth read out reflected the AR status (Figure 4B and 4C). Conversely, ectopic expression of *NEDD4* in *PMEPA1* shRNA stable transfectants was refractory to AR biochemical signals and associated features (Figure 4D). These findings established that both PMEPA1 and NEDD4 are essential in AR mediated cell growth.

### NEDD4 down-regulates AR protein levels and inhibits cell growth in hormone responsive prostate cancer cells

**Figure 5:**
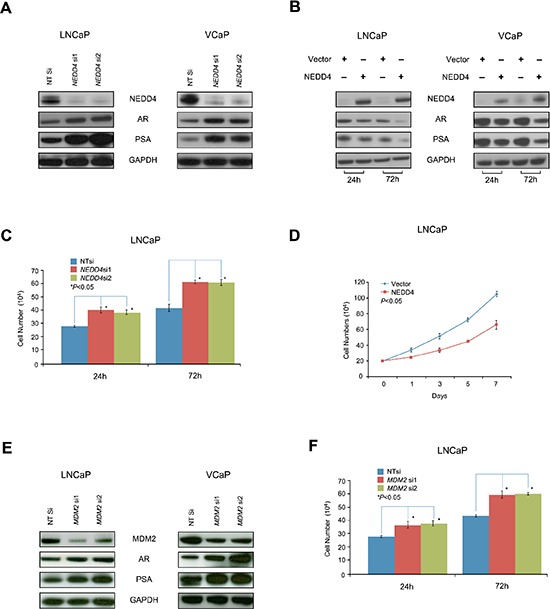
NEDD4 down-regulates of AR protein level and AR signaling, as well as inhibits the growth of LNCaP and VCaP cells **(A)** Inhibition of *NEDD4* by siRNA (*NEDD4* si1 and si2) enhances AR and PSA protein levels in LNCaP and VCaP cells. **(B)** Ectopic expression of *NEDD4* decreases AR and PSA in LNCaP and VCaP cells. **(C)** Cell counting assay reveals that *NEDD4* siRNA (*NEDD4* si1 and si2) increases the growth of LNCaP cells (*p* < 0.05). **(D)** Conversely, ectopic expression of *NEDD4* inhibits the growth of LNCaP cells (*p* < 0.05) as shown by cell counting assay. **(E)** As expected, *MDM2* siRNA (*MDM2* si1 and si2) also increases AR and PSA protein levels in LNCaP and VCaP cells.

Our previous study has revealed that PMEPA1 binds to NEDD4 through the interaction between PY motifs of PMEPA1 and WW domain of human NEDD4 protein [17]. Thus, we evaluated the role of NEDD4 on CaP cell growth and AR protein levels. Knockdown of *NEDD4* by specific siRNA led to strikingly elevated levels of AR and PSA in LNCaP and VCaP cells (Figure 5A). Conversely, ectopic expression of the *NEDD4* resulted in decreased levels of AR protein in both LNCaP and VCaP cells (Figure 5B). Further, ectopic expression of *NEDD4* resulted in a significant inhibition of cell growth (Figure 5C and 5D). In parallel, we also evaluated the effects MDM2 E3 ligase on AR which has been reported to be involved in AR degradation [15]. As expected, transfection of the *MDM2* siRNA, resulted in elevated AR and PSA protein levels in both LNCaP and VCaP cells (Figure 5E and 5F). Taken together, these findings underscored that NEDD4 mediates AR degradation and cell growth inhibition in hormone responsive VCaP and LNCaP cells.

### NEDD4 facilitates PTEN degradation in *PMEPA1* depleted cells

**Figure 6:**
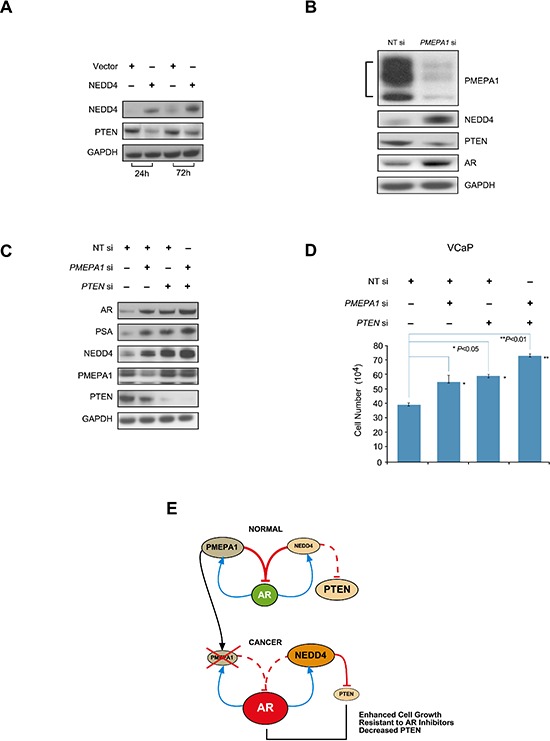
NEDD4 facilitates PTEN degradation in *PMEPA1* depleted cells **(A)** Western blot assay shows that overexpression of NEDD4 decreases PTEN in VCaP cells at 24 and 72 hours post transfection. **(B)** Inhibition of *PMEPA1* by siRNA upregulates NEDD4 and AR, and results in decreases in PTEN protein levels in VCaP cells. **(C)** Simultaneous knockdown of *PMEPA1* and *PTEN* leads to elevated AR and PSA levels and **(D)** enhanced cell growth (*p* < 0.01). **(E)** Model depicting three major consequences of PMEPA1 depletion in CaP cells such as increased cell growth, resistances to AR inhibitors and decrease PTEN protein level.

Genomic deletions and reduced expression of *PTEN* is frequently noted in CaPs which correlates with adverse pathologic features [36]. Ubiquitin-mediated degradation of PTEN by NEDD4 has been reported [37, 38]. As noted, above *PMEPA1* depletion in CaP cells also resulted in elevated NEDD4 levels. Thus, we reasoned that decreased *PMEPA1* may contribute to reduced levels of PTEN, a known NEDD4 target in CaP cells. In VCaP cells harboring wild type PTEN, ectopic expression of *NEDD4* down-regulates PTEN protein levels (Figure 6A). Moreover, the *PMEPA1* knockdown led to elevated levels of AR protein, increased NEDD4 and decreased PTEN protein levels (Figure 6B). These findings suggest that loss of *PMEPA1* may contribute to the defects of PTEN and alterations in NEDD4 through AR that is consistent with reported associations of PTEN and AR with CaP progression [36]. Thus, PMEPA1 may in part also affects NEDD4 mediated PTEN degradation in the context of androgen responsive CaP cells. However, NEDD4 can target PTEN independent of AR or PMEPA1. Ectopically expressed PTEN directly interacting with AR, has been shown to facilitate AR degradation and to inhibit AR nuclear translocation in PTEN null LNCaP cells [39]. Due to this reciprocal relationship between AR and PTEN, we evaluated the cooperative effects of *PTEN* knockdown and *PMEPA1* depletion on cell growth, AR levels and AR transcriptional targets. Transfection of VCaP cells with *PMEPA1* or *PTEN* siRNA resulted in enhanced cell growth, increased levels of AR and its targets, PSA and NEDD4. Cells depleted in both PMEPA1 and PTEN led to further increases in AR and cell growth (Figure 6C and 6D). Cumulatively, loss or reduced expression of *PMEPA1* may lead to higher levels of AR and decreased levels of PTEN resulting in a feedforward enhancement in cell growth as shown in simultaneous depletion of PMEPA1 and PTEN.

### Higher ratio of *PSA* to *PMEPA1* expression as a surrogate for increased AR function associates with more aggressive prostate cancer cells

The frequently observed silencing of *PMEPA1* in CaP may result in stabilization of AR, leading to higher levels of AR and increased AR transcription factor activity. To assess this finding in human CaP specimens, laser capture micro-dissected matched benign and cancer epithelium derived RNA samples were evaluated for quantitative expression of *PMEPA1* and PSA/*KLK3* mRNA, a surrogate for AR transcriptional activation function, in frozen radical prostatectomy derived specimens from 82 patients. The experiment revealed a correlation of decreased *PMEPA1* mRNA expression with increased PSA/*KLK3* expression levels. The ratio of *PSA* and *PMEPA1* T/N expression values in each patient was correlated with clinico-pathological characteristics. Higher *PSA/PMEPA1* expression ratio significantly associated with multiple indicators of disease progression (tumor differentiation, margin status, extracapsular extension and seminal vesicle invasion (Table 3). Thus, increased AR function as reflected by increased PSA/*KLK3* expression along with reduced *PMEPA1* expression associate with progressive CaP.

**Table 3:** Assessment of AR activity by PSA/*KLK3* gene expression relative to *PMEPA1* showed association of increased PSA(*KLK3*)/*PMEPA1* ratios with poor clinicopathologic features

Clinicopathologic Characteristics		Log ratio PSA*(KLK3)/PMEPA1* T/N		
		*N*	Mean (SD)	*P* value
LCM Tumor Differentiation	Well	68	0.52 (1.37)	0.0218
	Poorly	14	1.52 (1.80)	
Margin status	Negative	66	0.5 (1.4)	0.0293
	Positive	16	1.3 (1.6)	
Extra Capsular Extensions (ECE)	Negative (pT2)	47	0.4 (1.4)	0.0251
	Positive (pT2–3)	34	1.1 (1.5)	
		PSA*(KLK3)/PMEPA1* (T) Ratio Fold Change		
Seminal Vesicle (SV) Invasion	Negative	71	0.006	0.0063
	Positive	11	2.828	

## DISCUSSION

It is well recognized that the alterations (gain or loss) of the AR mediated signaling, play critical roles in the development and progression of CaP. While AR has been extensively studied for alterations of its structure, expression and transcriptional functions in CaP, defects of the AR protein degradation in CaP warrant careful evaluations. Better understanding of mechanisms controlling AR protein levels may also help in developing new therapeutic strategies focusing on AR degradation in a majority of metastatic CaP associating with the gain of AR function.

Cumulative data underscore the association of reduced or absent *PMEPA1* expression in CaP cells with increased AR due to impaired AR degradation [17–19]. However, the impact of attenuated *PMEPA1* expression in the interface of NEDD4, AR and PTEN in CaP biology and the effects on therapeutic AR inhibitors has not been examined before. Three major consequences of loss of *PMEPA1* in CaP are summarized in the working model (Figure 6E). First, *PMEPA1* knockdown accelerated the growth of LNCaP tumors in athymic nude mouse and in cell culture models. Second, *PMEPA1* knockdown resulted in enhanced resistance to both enzalutamide and bicalutamide inhibitors of AR. Third, we noted that *PMEPA1* silencing increased NEDD4-mediated downregulation of PTEN, further enhancing the growth of CaP cells. In agreement with cell culture models, in human CaP specimens decreased *PMEPA1* expression correlated with increased AR transcriptional function as measured by PSA/*KLK3* mRNA expression.

One of the new findings of this study is the androgen induction of NEDD4 E3 ligase that may impose additional level of hormonal control on AR and PTEN degradation (Supplementary Figure 3A–3C). Previous studies reported androgen induction of *NEDD4L* transcripts [40]. Informatics analysis of regulatory elements in the *NEDD4* gene locus indicated the presence of a cluster of androgen responsive elements (Supplementary Figure 3D).

Of note, this study also provides novel findings of cooperation between *PMEPA1* and *PTEN* defects leading to enhanced CaP cell growth. While *PMEPA1* depletion uncouples NEDD4 from AR degradation, increased levels of NEDD4 remains available for PTEN degradation, leading to simultaneous gain of AR and loss of PTEN in CaP cells. Evaluating the mechanism of *PMEPA1* silencing in previous studies we have shown that methylation of the first intronic DNA region of *PMEPA1* gene is a prevalent mechanism of *PMEPA1* silencing in human prostate CaP [41]. In conclusion, this study highlights that silencing of *PMEPA1* accelerates the growth of CaP cells through AR, NEDD4 and PTEN. Taken together, these data provide support for the dual impact of PMEPA1 restoration as a complementary approach in correcting for AR and PTEN defects in CaP and highlights biomarker potential of monitoring *PMEPA1* during androgen ablation therapy.

## MATERIALS AND METHODS

### Cell culture and related reagents

LNCaP and VCaP cells (from ATCC, Manassas VA) were maintained in RPMI 1640 and DMEM growth medium, respectively, supplemented with 10% fetal bovine serum (FBS). For the androgen-depletion experiments, cells were grown in 10% charcoal stripped FBS supplemented cell growth medium for 5 days. Subsequently, cells were treated with various dosages (0 nM, 0.1 nM, 1.0 nM and 10.0 nM) of synthetic androgen R1881 (Cat#NLP005005MG, Perkin Elmer Life Science, Waltham, MA) for indicated time. Hexamethrine bromide (Cat#H9268–5G) and puromycin (Cat#A1113802) used for selection *PMEPA*1 shRNA lentivirus transfectants were purchased from Sigma-Aldrich (St. Louis, MO).

### Plasmids, small interfering (si) RNAs and shRNA lentiviruses

The mammalian plasmids used were as follows: pcDNA3.1 as vehicle control, pcDNA3.1-*PMEPA1*, pCMV-AR (wild-type) and pCMV-AR (mutant T877A) were described before (15, 37); pCMV-XL4 (vehicle control, Cat#PCMV6XL4) and pCMV-XL4-*NEDD4* (Cat#SC107811) were purchased from OriGene (Rockville, MD). The siRNAs for various genes were all purchased from Dharmacon (Lafayette, CO). Each gene of interest was targeted with two different siRNAs (Supplementary 4A–4C) using optimal concentrations that were determined in pilot assays: *PMEPA1* siRNA1: GTTATCACCACGTATATA; *PMEPA1* siRNA2: 5′-GCATCAGCGCCACGTGCTA-3′; *NEDD4* siRNA1: 5′-TGCAGAACAGGCTGAGGAA-3′; *NEDD4* siRNA2 ATGAAACTTCGCCGAGCAA; *AR* siRNA1: 5′-GCAAAGGTTCTCTGCTAGA-3′; *AR* siRNA2: 5′-TCGAGGCCCTGTAACTTG-3′; *MDM2* siRNA1: 5′-GCCACAAATCTGATAGTA-3′; *MDM2* siRNA2: 5′-GAAGTTATTAAAGTCTGTT-3′; *PTEN* siRNA1: 5′-GTATAGAGCGTGCAGATAA-3′; *PTEN* siRNA2: 5′-GTTAGCAGAAACAAAAGGAGATATCAA-3′. The co-transfection was conducted with *PMEPA1* siRNA1, *AR* siRNA2 and *PTEN* siRNA2. The non-targeting control siRNA pool (Cat#D-001206–13–50) was also purchased from Dharmacon (Lafayette, CO). The Mission shRNA lentiviral transduction particles (Non-Target, Cat#SHC002V and *PMEPA1*, Cat#SHCLNV and Clone ID: TRCN0000272494) were purchased from Sigma-Aldrich (St. Louis, MO). The targeting sequence of *PMEPA1* shRNA1 was 5′-GAGCAAAGAGAAGGATAAACA-3′, and *PMEPA1* shRNA2 was 5′-GAGTTTGTTCAGATCATCATC-3′. The transfection of plasmids or small interfering RNA were performed following the standard protocol of lipofectamine 2000 (Life Technology, Carlsbad, CA). The infection of mission shRNA lentiviruses transduction particles and selection of positive colonies were performed as recommended by the supplier.

### Cell counting assays

The CaP cells (LNCaP and VCaP cells) were seeded in 6 cm culture dishes at the density of 2 × 10^5^ cells/dish. Cells were grown at 37°C, 5% CO_2_ for 2 days followed by transfection with plasmids (pcDNA3.1, pcDNA3.1-*PMEPA1*, pCMV-XL4, pCMV-XL4-*NEDD4* or pCMV-XL4-*AR*) or siRNA (non-targeting, *PMEPA1*, *AR*, *NEDD4* or *PTEN* siRNA) using lipofectamine 2000 procedure. The AR inhibitors treatment experiments were conducted with *PMEPA1* shRNA1 harboring LNCaP and VCaP cells. The transfected cells or the stable transfectants were harvested at indicated time points post-transfection with 0.25% trypsin plus EDTA (Life Technology, Carlsbad, CA). Cells were re-suspended into 10 ml regular medium, and 10 μl of single cell suspension was applied to hemocytometer for cell counting with trypan blue (Cat#72–57-1, Sigma-Aldrich, St. Louis, MO).

### Cell cycle analysis by fluorescence activated cell sorting

Ten million cells of *PMEPA1* shRNA transfectant LNCaP or VCaP cells were harvested by centrifugation at 180 × g for 5 min. Cells were resuspended in 300 μl PBS (pH 7.4), and 5 ml methanol was added drop-wise and vortexed at low speed. The fixed cells were incubated in methanol at −20°C for 30 min. After removing the methanol cells were stained by propidium iodide dye (Sigma-Aldrich, St. Louis, MO) at final concentration of 60 μg/ml containing 50 μg/ml RNaseA in PBS at room temperature (RT) for 30 min and were processed for fluorescence-activated cell sorting (FACS) analysis.

### Western blot analysis

The cells were harvested with M-PER mammalian protein extraction reagent (Cat#78501, Thermo Scientific, Rockford, IL) supplemented with protease inhibitor mixture (Cat#P8340, Roche Applied Science, Indianapolis, IN) and phosphatase inhibitor mixture (Cat#P-0044, Sigma-Aldrich, St. Louis, MO). Cell lysates were analyzed using Western blot procedures described in details in the Supplementary Methods.

### Antibodies

Antibodies used for Western blot assay were as follows: anti-AR rabbit polyclonal antibody (N-20, Cat#sc-816, Santa Cruz Biotechnology, Santa Cruz, CA); anti-PMEPA1 mouse monoclonal antibody (2A12, Cat#H00056937-M01, ABNOVA, Taiwan); anti-NEDD4 rabbit polyclonal antibody (Cat#07–049, Millipore, Billerica, MA); anti-PTEN mouse monoclonal antibody (Cat#MS-1601-S0, Lab Vision, Fremont, CA), anti-PSA rabbit polyclonal antibody (Cat#A05662, Dako, Denmark); anti-GAPDH polyclonal antibody (FL335, Cat#sc-25778, Santa Cruz Biotechnology, Santa Cruz, CA); and horseradish peroxidase (HRP)-conjugated anti-rabbit and anti-mouse TrueBlot antibodies (Cat#18–8816, Cat#18–8817-33, eBioscience, San Diego, CA). Antibodies for immunohistochemistry (IHC) assay were: anti-PMEPA1 antibody mentioned above, anti-AR mouse monoclonal antibody (M3562, DAKO, Denmark), anti-PSA rabbit polyclonal antibody (Cat#A0562, DAKO, Denmark), biotinylated horse anti-mouse antibody (Cat# BA-2000, Vector, Burlingame, CA), biotinylated goat anti-rabbit antibody (Cat# BA-1000, Vector, Burlingame, CA) and VIP peroxidase (HRP) substrate kit (Cat# SK-4600, Vector, Burlingame, CA).

### Athymic nude mouse tumorigenicity assay

Ten weeks old athymic Ncr-nu/nu mice (National Cancer Institute, Frederick, MD) were evaluated in tumor xenografts derived from *PMEPA1* shRNA LNCaP and control-shRNA LNCaP cells. Mice were maintained under pathogen-free conditions in accordance with established NIH guideline under the animal protocol approved by Uniformed Services of University of the Health Sciences, Institutional Animal Use and Care Committee. A total of 40 athymic nude mice were subdivided into two groups: control-shRNA LNCaP and *PMEPA1*-shRNA LNCaP with 20 mice in each group. Transfectant cells were resuspended in RPMI1640 medium mixed with 50% matrigel (Cat#354234, BD Biosciences, Bedford, MA). Each mouse received 4 × 10^6^ cells in 200 μl medium by subcutaneous injection to right frank side. Injected mice were monitored for tumor formation twice per week post injection. The end point of the experiment represented tumor diameter of 1.5 cm or 6 weeks after xenograft tumor formation. The excised tumor tissue was fixed in 10% formalin, paraffin-embedded and sectioned for 5 μm slides for Hematoxylin and Eosin stain and immunohistochemistry (IHC) staining for PMEPA1, AR and PSA. Total 10 foci with 20X magnification were randomly examined from each IHC staining slides for quantitative analysis with ImageJ software (version 1.48, National Institutes of Health, Bethesda, MD). The IHC procedures were described in details in the Supplementary Methods.

### Quantitative RT-PCR

Quantitative RT-PCR was performed using RNA laser capture microdissected matched normal and prostate tumor specimens as described in Sterbis *et al* [42]. PCR primers and Taqman probes were as follows: *PMEPA1*, 5′-CATGATCCCCGAGCTGCT-3′ (forward), 5′-TGATCTGAACAAACTCCAGCTCC-3′ (reverse), and 6FAM-5′-AGGCGGACAGTCTCCTGCGAAACC-3′-TAMRA (probe); PSA/*KLK3*, 5′-CCCACTG CATCAGGAACAAA-3′ (forward), 5′-GAGCGGGTGTG GGAAGCT-3′ (reverse), and 6FAM-5′-ACACAGGCCAG GTATTTCAGGTCAGCC-3′-TAMRA (probe). The expression of *GAPDH* served as an endogenous control measured in the same tube as the target gene (*GAPDH* control mix, Applied Biosystems). Target gene expression in each sample was normalized to *GAPDH*. Results were plotted as average Ct (cycle threshold) values of duplicate samples. Relative gene expression level was presented as dCt in tumor versus matched benign cells, where dCt represents normalized Ct value of target genes to *GAPDH*. Statistical analysis of quantitative expression of *PMEPA1* and PSA mRNA was evaluated in 82 patients (164 tumor and matched normal samples). The sample size (*N* = 82) was determined by statistical power calculation (90% power with 0.05 alpha, two sided Chi-square test). Distributions of other clinico-pathological variables were examined by using Chi-square or Fisher exact tests.

### Statistical analysis

If not otherwise stated, significant was calculated utilizing an unpaired *t*-test. Data are calculated as mean ±SEM or +SEM.

## SUPPLEMENTARY METHODS AND FIGURES


