# Supplementary material for: Silencing of PMEPA1 accelerates the growth of prostate cancer cells through AR, NEDD4 and PTEN
Source: Oncotarget. 2015 Mar 30;6(17):15137–49. doi: 10.18632/oncotarget.3526 (PMC4558141; doi:10.18632/oncotarget.3526)
Supplement: Supplementary file 1 [file oncotarget-06-15137-s001.pdf]

SUPPLEMENTARY METHODS

Cell lysis and western blot

The lysed cells were kept on ice for 30 min while vortexing at high speed for 15 second every 15 min. After centrifugation at 10,000 ×g at 4°C for 10 min, the supernatants were transferred into the pre-chilled Eppendorf tubes as total protein. The protein concentration in cell lysates was measured using Protein Assay Reagent from Bio-Rad (Hercules, CA). Cell lysates equivalent to 20 μg of the total protein were loaded on to NuPAGE 4–12% Bis-Tris Gel (Invitrogen by Life Technology, Carlsbad, CA) for electrophoresis at 130 V until the sample loading dye band reached the bottom of NuPAGE gel. Electrophoresed proteins from the gel were transferred to PVDF membrane (Invitrogen by Life Technology, Carlsbad, CA) at 30 V for 70 min. The PVDF membrane with transferred proteins was washed with 0.1% PBST for 5 min followed by blocking with 5% frat-free milk (Bio-Rad, Hercules, CA) in 1 × Dulbecco's Phosphate Buffered Saline (DPBS) (GIBCO, Auckland, NZ) for 30 min at room temperature. The membrane was incubated with primary antibody overnight. The membrane was washed three times with 0.1% PBST for 5 min. The secondary antibody was incubated for 1 hour at room temperature. After three washes with 0.1% PBST for 5 min, the membrane was treated with Amersham ECL Western Blotting Detection Reagent (GE Healthcare, Buckinghamshire, UK) for 1 min. The film was exposed to the membrane and developed.

BrdU incorporation assay

The 5-bromo-2′-deoxy-uridine (BrdU) labeling and detection kit (Cat#11296736001) was purchased from Roche Applied Science (Indianapolis, IL). *PMEPA1* shRNA lentivirus transfectant LNCaP and VCaP were plated into 6 cm dishes at the density of 2 × 10^5 cells/dish. The cells were treated with BrdU labeling reagent (10 μM) for 1 hour, harvested for cytospin slides and processed as described by the supplier (Roche Applied Science, Indianapolis, IL).

Cell plating efficiency and soft agar colony formation assay

The *PMEPA1* shRNA stable transfectants of LNCaP cells were plated in 6-well cell culture plates at the density of 1000 cells/well or 5000 cells/well. The cells were cultured at 37°C, 5% CO_2 for 10 days without re-feeding the medium. The colonies were harvested with 10% formalin and stained with 1% crystal violet. Soft agar (Cat#214220) was purchased from BD Biosciences (San Jose, CA). Each well of a 6-well plate was loaded with 2 ml 0.5% agar as base layer first, and 2 ml 0.3% agar top layer mixed with the stable transfectant LNCaP cells (0.5 × 10^4 cells and 5 × 10^4 cells/ml). Colonies were counted after 2 weeks.

Immunohistochemisty (IHC) assay

The formalin fixed and paraffin embedded slides were deparaffinized with Xylene and rehydrated 100% ethanol. The slides were treated with 0.6% hydrogen peroxide in 100% methanol to block endogenous peroxidase for 20 min at room temperature (RT). After microwaving antigen retrieval in EDTA buffer (pH8.0) for 25 min, the slides were blocked with 1% normal horse (for AR and PMEPA1 antibody) or goat serum (for PSA antibody), the primary antibodies were applied for 60 min at RT. After rinsing with PBS (pH7.4), the slides were incubated with related secondary antibodies in 5% nonfat dry milk/PBS for 20 min at RT. The ABC kit (Cat#pk-6100) and VIP Chromogen substrate kit (Cat#SK-4600) purchased from Vector (Burlingame, CA) were applied according to manufacturer's protocol. The slides were further counterstained with Mayer's hematoxylin for 1 min at RT.

SUPPLEMENTARY FIGURES

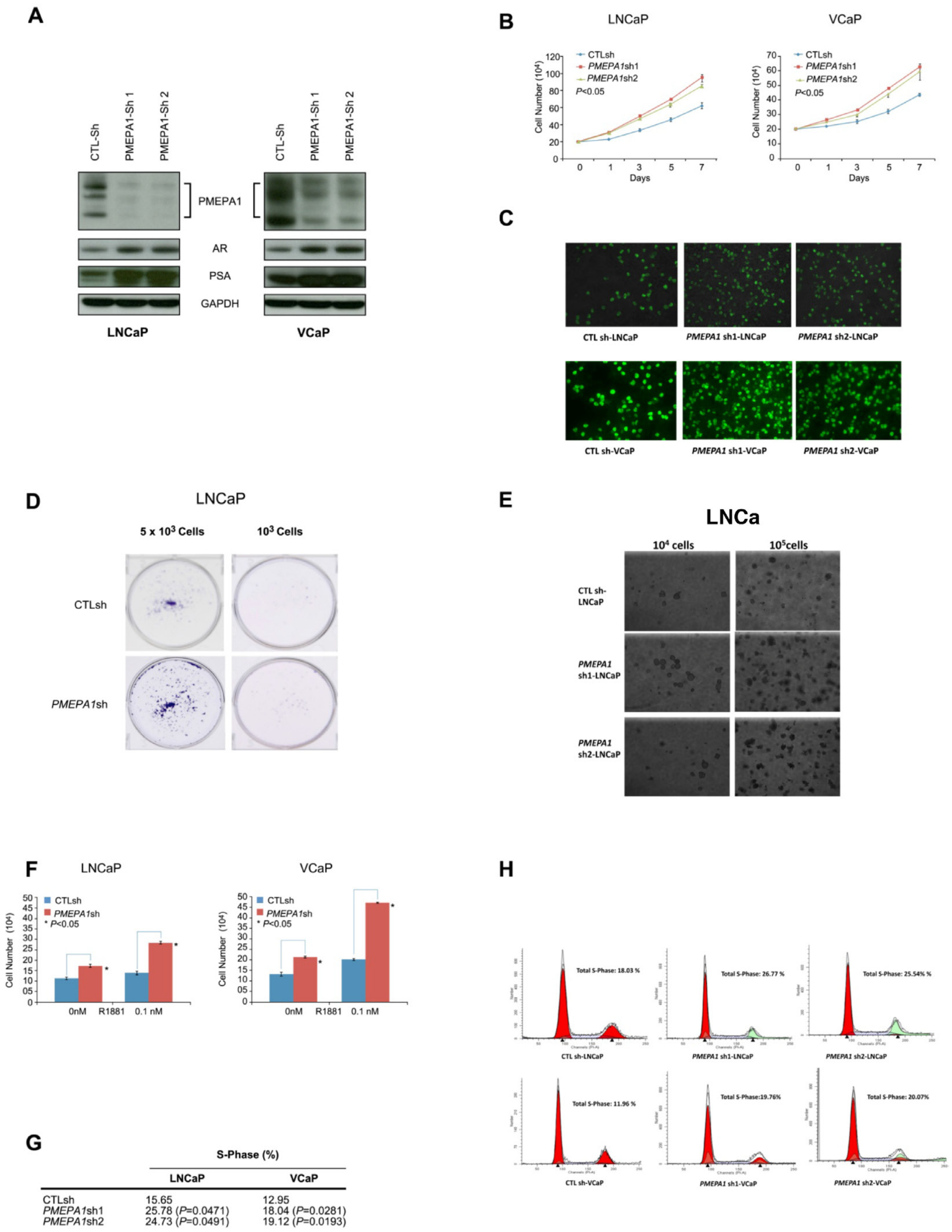

Supplementary Figure 1: (1A), Western blot assay demonstrated that decreased PMEPA1 and increased AR and PSA protein levels in stable transfectants harboring *PMEPA1*shRNA (*PMEPA1* sh1 and 2) compare to control shRNA (CTLsh) harboring transfectant in LNCaP and VCaP cells. (**1B** and **1C**), LNCaP and VCaP cells harboring *PMEPA1* shRNA (*PMEPA1*sh 1 and 2) exhibited higher cell growth rates (1B) and increased BrdU incorporation (1C, 40× magnification) in contrast to control cells. **(1D)**, cell plating efficiency assay showed *PMEPA1* shRNA (*PMEPA1* sh) harboring LNCaP cells formed more colonies at plating density of both 1000 and 5000 cells. **(1E)**, *PMEPA1*shRNA harboring LNCaP cells (*PMEPA1* sh) formed more colonies in 0.3% soft agar. **(1F)**, cell number of PMEPA1shRNA harboring LNCaP (left panel) and VCaP (right panel) cells were further increased in response to androgen (R1881) treatment. **(1G and 1H)**, FACS assay showed that LNCaP and VCaP cells harboring *PMEPA1*shRNA (*PMEPA1* sh) show higher percentages of cells in "S"-phase of cell cycle. ($P < 0.05$, *t*-test).

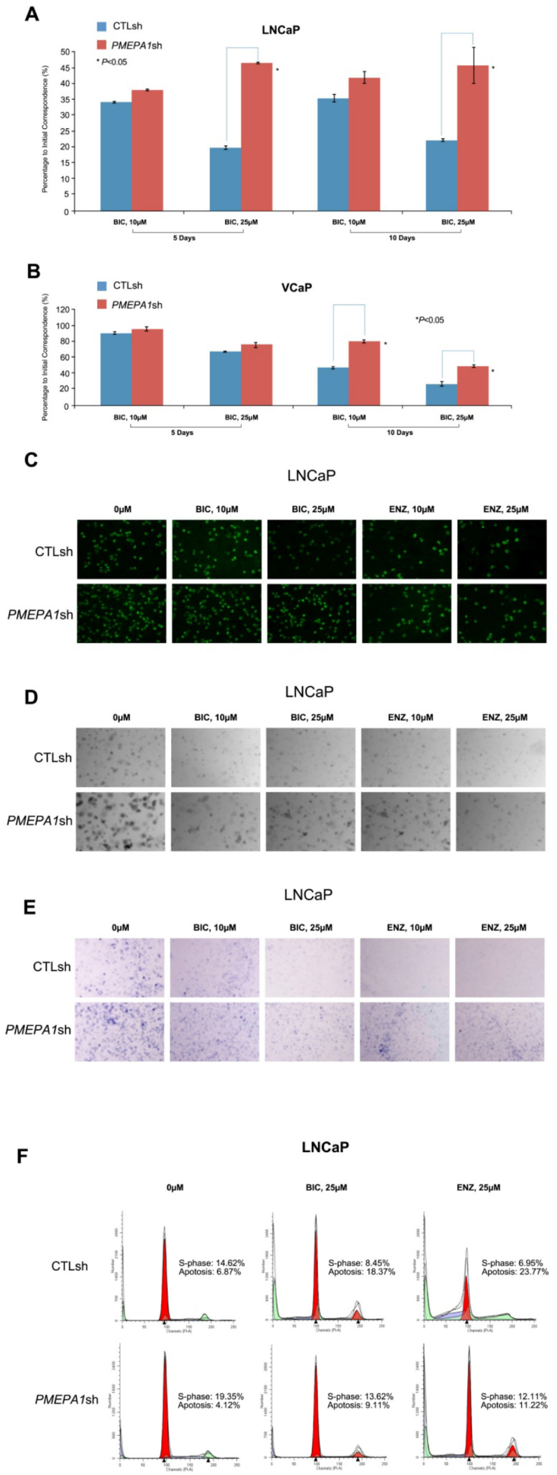

Supplementary Figure 2: (2A and 2B), cell counting assay showed *PMEPA1*shRNA (*PMEPA1* sh) harboring LNCaP and VCaP cells had higher percentages to initial cell numbers (2 × 10^5) compared to control in response to enzalutamide (ENZ) biocalutamide (BIC) treatment at dosages of 10 or 25 µM for 5 or 10 days (*P* < 0.05, *t*-test). (2C–2E), compared to control, *PMEPA1* shRNA (*PMEPA1* sh) harboring LNCaP cells have stronger BrdU incorporation (2C), increased soft agar colony formation (2D) and enhanced cell plating efficiency (2E) post in response to AR inhibitors treatment (ENZ and BIC) treatment at dosages of 10 or 25 µM for 5 days (BrdU incorporation assay) or 10 days (cell plating efficiency assay and soft agar colony formation assay). **(2F)**, FACS assay showed that more percentage of cells in S-phase and less apoptotic cells were detected in *PMEPA1*shRNA harboring LNCaP cells (*PMEPA1* sh) in response to AR inhibitors (ENZ and BIC) treatment at dosages of 25 µM for 5 days.

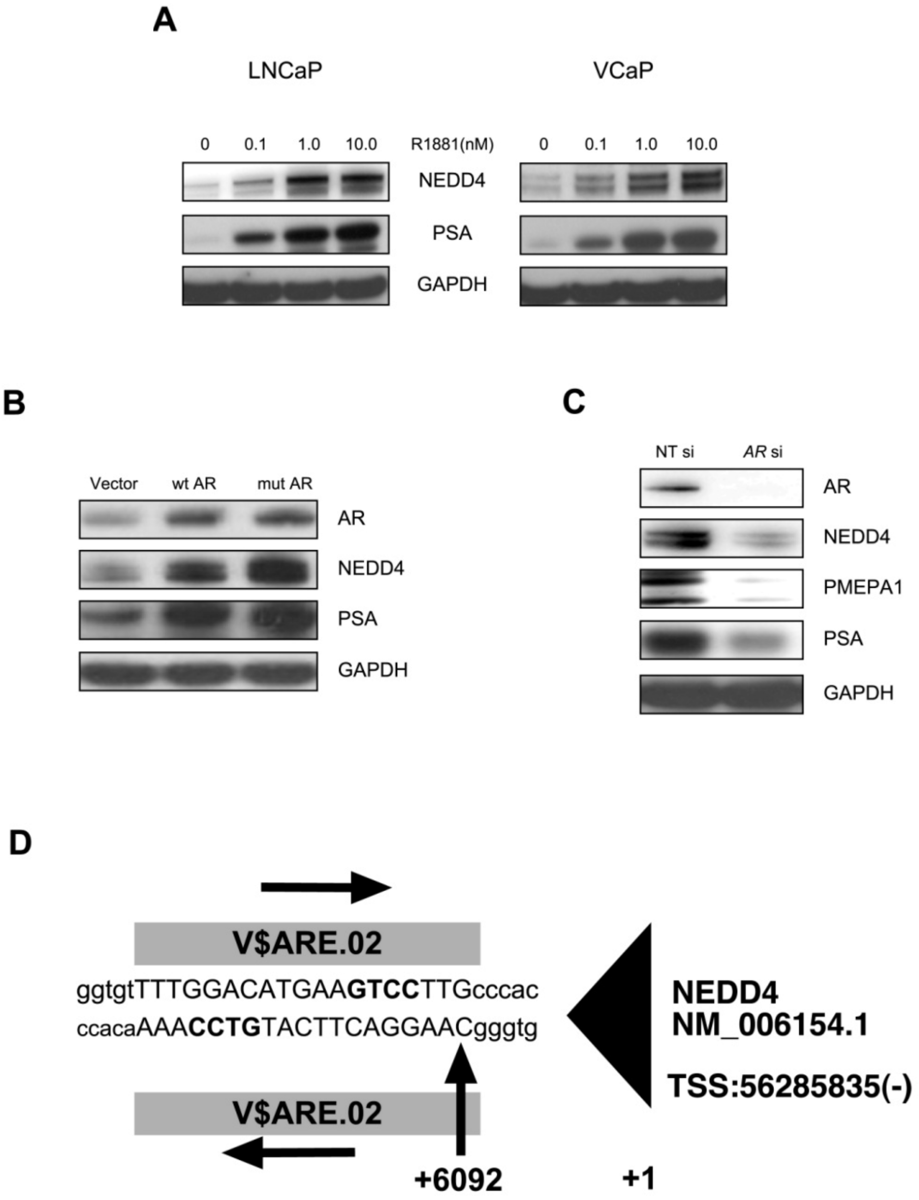

Supplementary Figure 3: (3A), Western blot assay shows androgen (R1881) dose dependent induction of NEDD4 protein in both LNCaP and VCaP cells. (3B), ectopically expressed wide type (wt) or T877A mutant (mut AR) AR upregulated protein levels of NEDD4 and PSA in LNCaP cells. **(3C)**, *AR* siRNA (*AR*Si) decreases NEDD4, PMEPA1 and AR protein levels in LNCaP cells. **(3D)**, two lower affinity AR binding sites in opposite polarity form a cluster of predicted AR-responsive element (V$ARE.02) on the *NEDD4*–1 gene promoter downstream region. Triangle marks the transcription start site (TSS) of the transcript at absolute position 56285835 on the negative strand(-) of Homo sapiens chromosome 15q21.3 (NCBI build 37). Capital letters indicate the predicted binding site with underlined core recognition sequences.

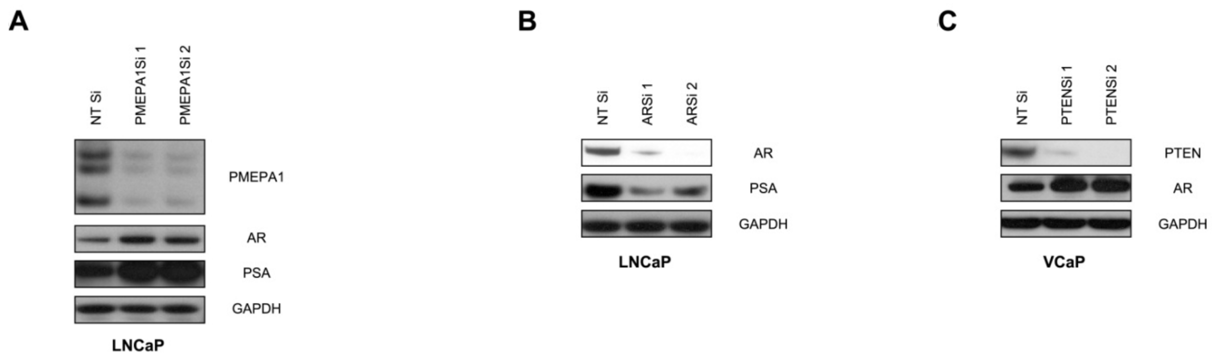

Supplementary Figure 4: (4A), Western blot assay showed that decreased protein level of PMEPA1 and enhanced protein levels of AR and PSA in LNCaP cells transfected with both *PMEPA1*siRNA1 and 2. (4B), decreased protein levels of AR and PSA were detected in LNCaP cells transfected with *AR* siRNA1 and 2 with Western blot assay. **(4C)**, Western blot assay revealed that suppressed protein level of PTEN and increase AR protein level in VCaP cells transfected with *PTEN* siRNA1 and 2.
